# Impact of peri-intraventricular haemorrhage and periventricular leukomalacia in the neurodevelopment of preterms: A systematic review and meta-analysis

**DOI:** 10.1371/journal.pone.0223427

**Published:** 2019-10-10

**Authors:** Juliana Wendling Gotardo, Nathalia de Freitas Valle Volkmer, Guilherme Pucci Stangler, Alícia Dorneles Dornelles, Betânia Barreto de Athayde Bohrer, Clarissa Gutierrez Carvalho

**Affiliations:** 1 Department of Pediatrics, Hospital de Clínicas de Porto Alegre, Porto Alegre, Brazil; 2 Faculty of Medical Sciences, Universidade Federal do Rio Grande do Sul, Porto Alegre, Brazil; 3 Department of Radiology, Hospital de Clínicas de Porto Alegre, Porto Alegre, Brazil; Centre Hospitalier Universitaire Vaudois, FRANCE

## Abstract

**Context:**

Whether all degrees of periventricular leukomalacia (PVL) and peri-intraventricular haemorrhage (PIVH) have a negative impact on neurodevelopment.

**Objective:**

To determine the impact of PVL and PIVH in the incidence of cerebral palsy, sensorineural impairment and development scores in preterm neonates. Registered in PROSPERO (CRD42017073113).

**Data sources:**

PubMed, Embase, SciELO, LILACS, and Cochrane databases.

**Study selection:**

Prospective cohort studies evaluating neurodevelopment in children born preterm which performed brain imaging in the neonatal period.

**Data extraction:**

Two independent researchers extracted data using a predesigned data extraction sheet.

**Statistical methods:**

A random-effects model was used, with Mantel-Haenszel approach and a Sidik-Jonkman method for the estimation of variances, combined with Hartung-Knapp-Sidik-Jonkman correction. Heterogeneity was assessed through the I^2^ statistic and sensitivity analysis were performed when possible. No funnel plots were generated but publication bias was discussed as a possible limitation.

**Results:**

Our analysis concluded premature children with any degree of PIVH are at increased risk for cerebral palsy (CP) when compared to children with no PIVH (3.4, 95% CI 1.60–7.22; 9 studies), a finding that persisted on subgroup analysis for studies with mean birth weight of less than 1000 grams. Similarly, PVL was associated with CP, both in its cystic (19.12, 95% CI 4.57–79.90; 2 studies) and non-cystic form (9.27, 95% CI 5.93–14.50; 2 studies). We also found children with cystic PVL may be at risk for visual and hearing impairment compared to normal children, but evidence is weak.

**Limitations:**

Major limitations were the lack of data for PVL in general, especially for the outcome of neurodevelopment, the high heterogeneity among methods used to assess neurodevelopment and the small number of studies, which led to meta-analysis with high heterogeneity and wide confidence intervals.

**Conclusions:**

There was no evidence supporting the hypothesis that PIVH causes impairment in neuropsychomotor development in our meta-analysis, but review of newer studies show an increased risk for lower intelligence scores in children with severe lesions, both PIVH and PVL. There is evidence to support the hypothesis that children with any degree of PIVH, especially those born below 1000 grams and those with severe haemorrhage, are at increased risk of developing CP, as well as children with PVL, both cystic and non-cystic.

## Introduction

The incidence of preterm births worldwide varies significantly, being as low as 5% in some European countries and as high as 18% in some places in Africa [1]. There seems to be an inverse relationship between country income and the rate of preterm deliveries, where countries with low and lower-middle income tend to have higher rates (usually above 10%) [1]. In Brazil, a developing country, historical data showed a steady increase in the rate of preterm deliveries since the year 1980, reaching rates of approximately 10% in the year 2000 [2] and 11.9% in 2012 [3]. In the United States, however, preterm birth delivery rates have remained somewhat stable over the last years, varying from 10.4% in 2007 to 9.8% in 2016 and 10.0% in 2017 [4].

Incidence of complications such as peri-intraventricular haemorrhage (PIVH) is considered high in preterm infants, especially those born with less than 32 weeks gestational age (GA) [5]. In these early preterm infants, a recent cohort study found an overall incidence of 36.2% of any PIVH, with Papile grade III and IV affecting around 7.1% of these newborns [5]. Incidence of periventricular leukomalacia (PVL), the most common form of ischemic brain injury in the newborn, is also higher in preterm infants. A recent systematic review described a high incidence of white matter abnormalities in preterm infants, estimated at 39.6% for infants born under 28 weeks GA, 27.4% under 32 weeks GA and 7.3% under 37 weeks GA. The main associated factors with PVL in this study were intrauterine infection, premature rupture of membranes and chorioamnionitis [6].

Regarding the neurological outcomes of preterms affected by these complications, a recent systematic review regarding neurodevelopment in children with PIVH found an increased chance of death or moderate-to-severe neurodevelopmental impairment in children with both mild (Odds Ratio [OR] 1.48, 95% CI 1.26–1.73) and severe (OR 4.72, 95% CI 4.21–5.31) PIVH [7]. Another systematic review found an increased risk for cerebral palsy (CP) in children with cystic PVL, with a prevalence of 86% in this group [8]. To investigate whether the degree of PIVH and PVL impact the risk for adverse outcomes, another systematic review found evidence that moderate or severe PIVH (Papile grades 3 or 4), with or without associated PVL, were predictors for CP in later years [9]. However, the role of these complications in specific components of neurodevelopment, such as neuropsychomotor development, neurosensorial disability and other motor deficits remains unclear, especially regarding mild stages of PIVH and non-cystic PVL.

Therefore, this systematic review and meta-analysis has the objective of summarizing evidence available on the relations between PIVH and PVL in neurodevelopment, categorizing these exposures into degrees of severity when possible. We reviewed prospective cohort studies including premature infants born under 37 weeks GA evaluated either by brain ultrasound (US) or magnetic resonance imaging (MRI) for the occurrence of PIVH and PVL who were followed for at least 12 months corrected age for the outcomes of interest (neuropsychomotor development, visual and hearing impairment and cerebral palsy).

## Methods

This meta-analysis was planned, conducted and reported as stated in the PRISMA [10] guidelines. The review protocol was registered at PROSPERO (registration number CRD42017073113). The institutional review board and ethics committee of Hospital de Clínicas de Porto Alegre approved the study under the code PJ20180319. No consent was necessary since it is a systematic review and meta-analysis.

### Studies

As defined *a priori*, the following criteria were considered for selection of studies. This review included only prospective cohort studies published in peer-reviewed journals between the years 1960 and 2019. Only studies with a minimum of 12 months corrected age follow-up were deemed eligible for inclusion. Case reports, narrative reviews, case series, dissertations and letters/editorials were excluded. Only studies that reported long-term neurodevelopmental outcomes for patients born under 37 weeks GA were included. Were excluded studies that included mixed populations (preterm and at term) and studies that followed only infants with abnormal US or MRI.

### Exposure

Were eligible for inclusion in this systematic review studies that reported or provided data regarding neurodevelopment in patients presenting one or both of the following:

Peri-intraventricular haemorrhage: defined as abnormal bleeding in the peri-intraventricular region with or without ventricular enlargement, detected by US or MRI. We made no restriction on whether PIVH was reported as a single entity or further categorized into severity scores, and also did not exclude based on the classification system used, since throughout the years several scores were created and some of them fell in disuse.

Periventricular leukomalacia: defined as changes in the signal intensity or echogenicity of periventricular white matter, both in cystic and non-cystic form, detected by US or MRI. We included studies regardless of the classification system used and whether or not the study subdivided PVL into cystic and non-cystic.

Were included only studies in which imaging was performed within the neonatal period (first 28 days of life). Presence of other abnormalities (e.g, central nervous system malformations), when not excluded in the final article analysis, were noted but did not disqualify the article for inclusion in this review.

### Outcomes

We included only studies that provided data for the following outcomes defined *a priori* and were considered initially for meta-analysis those that either presented measure of effect or provided enough data to allow its calculation.

#### Primary outcome

Neuropsychomotor development: The original research protocol planned to include only studies which utilized the Bayley scale. Studies that used other assessment tools will be described in the systematic review. For the purpose of meta-analysis this outcome was treated as a continuous variable.

#### Secondary outcomes

Severe visual impairment: Defined as blindness, partially sighted patient or corrected acuity less than 20/200.

Severe hearing impairment: Defined as deafness, reported profound neurosensory hearing loss, use of hearing device or severe alteration in standard testing.

Cerebral palsy: Were included studies that defined cerebral palsy as diplegia, hemiplegia, paraplegia, Gross Motor Function Classification System (GMFCS) level of at least 2 (on a scale from 1, mild impairment, to 5, severe impairment), severe alteration in other standard testing and severe limitation to deambulation.

### Review methods

#### Information sources and search strategy

The search strategy was planned in conjunction by authors after independent informal review of the literature. The search was performed in the following databases: PubMed, EMBASE, Cochrane Library, LILACS and SciELO. Were included studies published until august 20, 2017. An additional search with the same strategy was performed including studies published from august 21, 2017 to april 20, 2019. The details of the terminology and specific search strategy used in each database are available in supplemental material (S1 File).

### Data collection and extraction

Two authors (J.W.G. and N.V.) independently performed the database searches. After unification of the resulting articles, both authors reviewed and excluded duplicates. This final database was then reviewed by both using titles and abstracts to further narrow articles of interest. Finally, all remaining articles were read in full length and scrutinized for information matching the inclusion criteria in this review. In case of disagreement between the aforementioned authors, a third one (C.G.C.) reviewed the article in question and resolved discrepancies. Data extraction for the systematic review and for meta-analysis was performed independently by J.W.G. and G.P.S. using predetermined sheets.

### Risk of bias

Assessment of the risk of bias was performed at study level using an adapted version of the Newcastle-Ottawa Scale, recommended by the Cochrane initiative in the evaluation of risk of bias in observational studies and is available in supplemental material (S2 and S3 Files).

### Statistical analysis

The statistical analysis was performed in R (version 3.5.0, Vienna, Austria). Studies were grouped both by comparable exposure and outcome, and measures of effect were calculated when possible. We considered for meta-analysis only those studies which provided sufficient data and presented a clear and concise definition of PIVH and PVL, allowing aggregation with other studies. Effect in binary outcomes was calculated using relative risk and in continuous outcomes using absolute mean difference. A random effects model was used since we assumed heterogeneity from different study populations is likely present. A Mantel-Haenszel approach and Sidik-Jonkman estimators for tau-squared was used, since this strategy allows data analysis without continuity correction in the case of rare events. For those analysis, a sensitivity analysis was also performed whenever possible, including and excluding studies with zero events. This statistical strategy, as shown in previous research, may provide better estimates in such cases [11]. Statistical heterogeneity was assessed by the method of using the I^2^ values, presented with 95% confidence intervals. We also used a Hartung-Knapp-Sidik_jonkman method, which is proposed as a way to produce better estimation of variances, as well as leading to more conservative results. For analysis including more than 10 studies, a funnel plot was generated to assess for publication bias.

### Additional analysis

Premature newborns are a heterogeneous population and children born at different gestational ages might present different causes for PIVH and PVL, influencing outcomes. To address this concern, an unplanned exploratory subgroup analysis dividing patients by weight ranks was performed for the outcome of cerebral palsy regarding patients with any degree of PIVH.

## Results

### Description of studies

**Fig 1:**
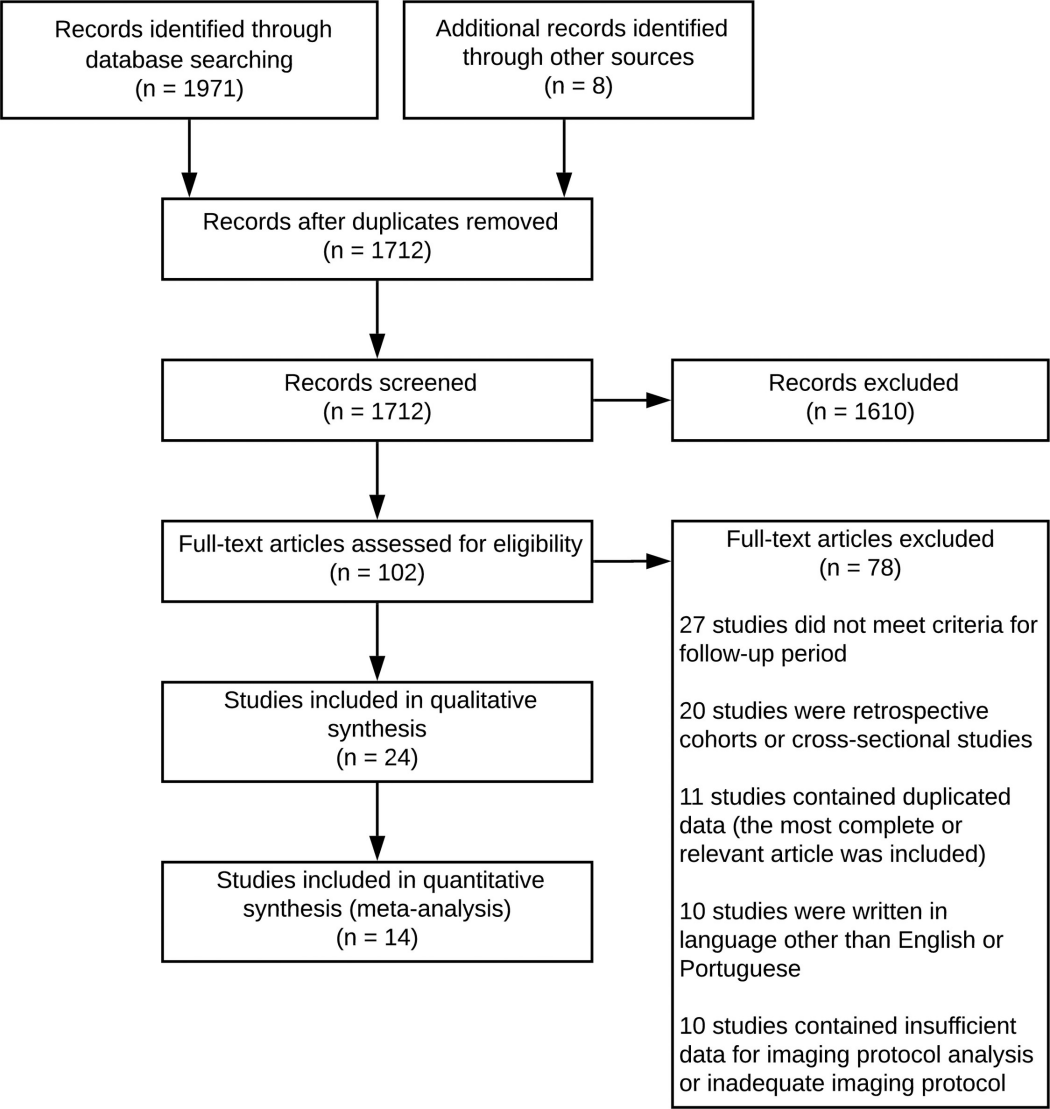
Flowchart of study selection.

From the initial 1971 references identified by systematic research and 8 references identified through secondary search, 102 were deemed eligible for full-text evaluation and resulted in 24 articles which met the inclusion criteria for this systematic review, as shown in Fig 1. The authors tried to reach original researchers whenever further clarification about the research was necessary or to provide missing data. Of the 24 articles included in the systematic review, 14 were used for meta-analysis, as shown in Table 1. The synthesized results of articles included in the systematic review is available in Table 2.

**Table 1:** Articles used in meta-analysis, sorted by outcomes with reasons for exclusion.

Outcome	Description of inclusion or exclusion in meta-analysis
**Cerebral Palsy**	**Number of included articles:** 24. **Included in meta-analysis:** 11 [12–15,19,21,22,25,28,33,34]. **Reasons for exclusion for meta-analysis:** Presented data for CP as combined outcome [16–18,20,23,26,31]. Data were not presented as binary outcome [24]. Measurement of exposure did not match any other [27,29,30,35]. Relevant imaging was not performed within criteria of inclusion [32].
**Hearing impairment**	**Number of included articles:** 15. **Included in meta-analysis:** 5 [15,23,25,26,34]. **Reasons for exclusion for meta-analysis:** No events in the cohort [12,14]. Not assessed [13,16,18,22,27,29–32]. Data were presented as combined outcome [17,20,21,28]. Outcome not assessed after 12 months [19]. Measurement of exposure did not match any other [24,33,34].
**Visual impairment**	**Number of included articles:** 16. **Included in meta-analysis:** 7 [12,14,23–26]. **Reasons for exclusion for meta-analysis:** Not assessed [13,16,18,22,27,29–32]. Data were presented as combined outcome [17,20,28,31]. Assessment method not described [13]. Outcome not assessed after 12 months [19]. Measurement of exposure did not match any other [33,35].
**Bayley I (MDI)**	**Number of included articles:** 9. **Included in meta-analysis:** 3 [12,13,14]. **Reasons for exclusion for meta-analysis:** Not sufficient data for analysis [16–18,21,25,32].
**Bayley I (PDI)**	**Number of included articles:** 9. **Included in meta-analysis:** 2 [12,13]. **Reasons for exclusion for meta-analysis:** Not sufficient data for analysis [13,16–18,21,25,32].

CP, cerebral palsy; MDI, mental development index; PDI, psychomotor development index.

**Table 2:** Results and characteristics of studies.

Author and year	Study population	Imaging and exposure	Follow-up	Main results
Catto-Smith [12], 1985	n = 31. GA between 23–28 weeks. Australia, 1981.	US: PIVH.	2 years corrected age.	Bayley: 3 children had MDI <50 (1 with IVH, 1 with ICH and 1 without PIVH).
Ment [13], 1985	n = 142. BW ≤1250 g. USA, June 1979 –June 1982.	CT and US: PIVH.	12, 18 and 30 months corrected age.	Blindness: 2 cases (one with and one without PIVH).
Szymonowicz [14], 1986	n = 32. BW ≤1250 g. Australia, 1982.	US: PIVH and PVL.	2 years corrected age.	Bayley: 3 infants had MDI <50 (1 with IVH, 2 with GLH+PVL). Major disability: 0/16 with normal US; 0/4 with GLH; 2/8 with IVH; 1/1 with ICH; 3/3 with PVL.
Graham [15], 1987	n = 156. BW ≤1500 g. UK, January 1984 –April 1985.	US: PVL and PIVH.	18 months corrected age.	Griffiths’ Median DQ: 108 with no US abnormality; 107 with any degree of PIVH; 109 with prolonged flare. Median DQ of the group with CP 92 (p = 0.012).
Salomon [16], 1987	n = 88. BW ≤1000 g. USA, January 1980 –June 1983.	US: PIVH.	1, 2 and 3 years chronological age.	Mild handicap: 21% with normal US; 27% with abnormal US. Severe handicap: 9% with normal US; 22% with abnormal US.
Nwaesei [17], 1988	n = 110. GA ≤32 weeks. Canada, July 1984 –June 1985.	US: PIVH and PVL.	12 months corrected age.	Mild Handicap: 20 cases. Severe/moderate Handicap: 19 cases (58% had major PIVH and/or PVL).
Bennett [18], 1990	n = 48. BW ≤1500 g or GA ≤32 weeks. USA, January 1983 –June 1984.	US: PIVH and PVL.	12–24 months corrected age.	Neurodevelopmental major abnormality (PIVH): 13% with no PIVH, 30% with PIVH Papile grade 1–2 and 44% with Papile 3–4. Neurodevelopmental major abnormality (PVL): 11% with no PVL, 25% with non-cystic PVL and 26% with cystic PVL.
Beverley [19], 1990	n = 62. GA ≤35 weeks. UK.	US: PIVH and PVL.	3, 6, 12 and 18 months corrected age.	Developmental delay: 7 cases (4 normal US, 1 cerebral atrophy, 1 Papile 3+PVL, 1 Papile 4). Blindness: 1 case (normal US).
Van de Bor [20], 1992	n = 31. GA ≤32 weeks. Netherlands.	US: PIVH and PVL.	3 years of age.	Minor Handicap: 3 cases (1 mental retardation, 1 minor neurological dysfunction and 1 with both), all with PIVH grade 3. Major Handicap: 6 cases (2 mental retardation, 2 CP, 2 with both), 5 with PVL and 1 with normal US.
Fazzi [21], 1992	n = 122. BW ≤1500 g. Italy, January 1983 –December 1987.	US: PIVH and PVL.	12 months corrected age.	Minor sequelae: 10 cases (4 normal US, 2 uncomplicated PIVH, 3 complicated PIVH, 1 PVL). Major sequelae: 26 cases (4 normal US, 3 uncomplicated PIVH, 14 complicated PIVH and 5 PVL). All children with complicated haemorrhage and PVL had CP.
Ikonen [22], 1992	n = 101. GA <33 weeks. Finland, May 1984 –May 1987.	US: PVL.	2 years of age.	Mental retardation: 7 cases, all with PVL. Visual disorders: 6 cases, all with PVL.
Van de Bor [23], 1993	n = 304. GA <32 weeks or BW <1500g. Netherlands, 1983.	US: PIVH.	5 years of age.	Neurological disability: 21 cases (13 no PIVH, 5 PIVH Papile grade 1–2 and 3 Papile 3–4). Neurological handicap: 16 cases (8 no PIVH, 5 PIVH Papile 1–2 and 3 Papile 3–4). Mental abnormality (disability): 37 cases (25 no PIVH, 10 PIVH Papile 1–2 and 2 Papile 3–4). Mental abnormality (handicap): 23 cases (15 no PIVH, 7 PIVH Papile 1–2 and 1 Papile 3–4). Language and Speech disability: 58 cases (40 no PIVH, 16 PIVH Papile 1–2 and 2 Papile 3–4). Language and Speech handicap: 22 children (16 no PIVH, 5 PIVH Papile 1–2 and 1 Papile 3–4).
Roth [24], 1993	n = 206. GA <33 weeks. UK, 1979–1982.	US: PIVH.	8 years of age.	Major neuromotor impairment: 13 cases (3 normal US, 1 uncomplicated PIVH, 2 ventricular dilatation, 1 hydrocephalus, 6 cerebral atrophy). Minor neuromotor impairment: 22 cases (14 normal US, 4 uncomplicated PIVH, 2 ventricular dilatation, 1 hydrocephalus, 1 cerebral atrophy). Wechsler Intelligence Scale for Children- revised (WISC-R) IQ <70: 13 infants (2 normal US, 3 uncomplicated PIVH, 3 ventricular dilatation, 1 hydrocephalus, 4 cerebral atrophy). WISC-R IQ 70–79: 7 infants (2 normal US, 1 uncomplicated PIVH, 3 ventricular dilatation, 1 hydrocephalus).
Aziz [25], 1995	n = 646. BW <1250 g. Canada, 1987–1990.	US: PIVH and PVL.	2 to 3 years of age.	Mental retardation: 15% of children with normal US; 24% of those with PIVH and 28% of those with cystic PVL.
Fawer [26], 1995	n = 295. GA ≤34 weeks. Switzerland, April 1982 –September 1986.	US: PIVH and PVL.	5 years of age.	Isolated visual impairment: 29% of children with small focal PVL. Neuromotor impairment: 9% of children with normal US, 12% of children with isolated PIVH and 21% of children with small PVL.
Vohr [27], 1999	n = 440. BW between 600–1250 g. USA, September 1985 –August 1992.	US: PIVH.	36 months corrected age.	Study compared early-onset PIVH and not early-onset PIVH. No data comparing US with PIVH versus normal scan.
Sherlock [28], 2005	n = 270. BW <1000 g or GA <28 weeks. Australia, January 1991 –December 1992.	US: PIVH.	8 years of age.	Neurosensory impairment: 28 cases with normal US, 5 cases with PIVH Papile grade 1, 5 cases with grade 2, 1 with grade 3, 6 with grade 4.
Dyet [29], 2006	n = 119. GA <30 weeks. UK, January 1997 –November 2000.	US and MRI: PIVH, PVL, diffuse excessive high signal intensity (DEHSI).	18 to 36 months corrected age.	Griffiths’ DQ (mean ±SD): No DEHSI 111±20; DEHSI 94±11.6; severe DEHSI 92±7.5; p = 0.23. PIVH and Punctate PVL did not predict lower DQ.
Vollmer [30], 2006	n = 567. GA <33 weeks. UK, 1979 and 1991.	US: PIVH.	8 years of age.	WISC-R Full-scale intelligence quotient (mean ±SD): Normal US 101 ±17 and PIVH with ventricular dilatation 96±23. CP: 2% of children with normal US and 16% of those with PIVH with VD.
Locatelli [31], 2010	n = 195. GA 24–32 weeks. Italy, January 1999 –December 2006.	US: PIVH and PVL.	24 months of age.	Adverse neurodevelopmental outcome (ANDO) defined as CP or neurodevelopmental delay. ANDO in 45 infants, severe in 28. 26 infants with ANDO had PIVH or PVL.
Van Wezel-Meijler [32], 2011	n = 130. GA <32 weeks. Netherlands, May 2006 –October 2007.	US and MRI: PIVH and PVL.	2 years of age.	PIVH and abnormal ventricle size or shape predicted outcomes at 2 years of age (positive predictive value 34 and 31%, respectively; negative predictive value 94%).
Klebermass- Schrehof [33], 2012	n = 151. GA <32 weeks. Austria, 1994–2005.	US: PIVH.	1, 2, 3 and 5 years of age.	Bayley II MDI <70 at 1 year: 9.7% of infants without PIVH, 7.6% PIVH Papile 1, 25.7% PIVH grade 2. PDI < 70 at 1 year: 15% no PIVH, 42.3% PIVH grade 1, 45.8% PIVH grade 2. KABC (Kaufmann’s assessment battery for children) <70 at 5.5 years: 5.2% without PIVH, 5.9% PIVH grade 1 and 10.5% PIVH grade 2. Visual impairment: 5.9% no PIVH, 17.9% PIVH grade 1, 21% PIVH grade 2. Acoustic impairment: 1.7% without PIVH, 2,5% with PIVH grade 2.
Payne [34], 2013	n = 1472. GA <27 weeks. USA, January 2006 –December 2008.	US: PIVH.	18–22 months corrected age.	Bayley III Cognitive score <70: 7% of infants without PIVH, 7% PIVH Papile 1 and 2 and 15% PIVH 3 and 4 (p value <0.01). Language score < 70: 16% no PIVH, 16% PIVH grade 1 and 2, 29% PIVH grade 3 and 4 (p value <0.01).
Hintz [35], 2018	n = 386. GA <28 weeks. USA, February 2005 –February 2009.	US: PIVH and PVL.	6 years and 4 months to 7 years and 2 months.	Bayley III Cognitive score < 70: 8% of infants without white matter abnormality (WMA), 11% in mild WMA, 12% in moderate WMA and 60% in severe WMA (p < 0.001). CP with GMFCS ≥ 2: none without WMA or with mild WMA, 2% in moderate WMA and 24% in severe WMA (p < 0.0001). Adverse US findings (PIVH 3 or 4, or cystic PVL: Increased risk of cognitive score < 70, cerebral palsy and mild or moderate-to-severe disability.

BW, birth weight; CT, computed tomography; DQ, development quotient; GLH, germinal layer haemorrhage; ICH, intracerebral haemorrhage; IQ, intelligence quotient; IVH, intraventricular haemorrhage; MDI, mental development index; PDI, psychomotor development index; SD, standard deviation; UK, United Kingdom; US, ultrasound; USA, United States of America; WMA, White matter abnormality.

### Neuropsychomotor development

**Fig 2:**
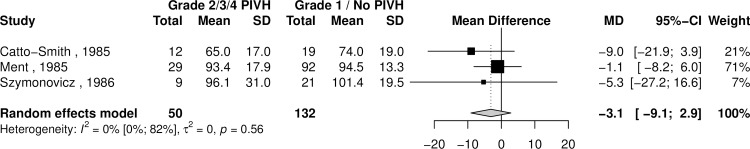
Absolute mean difference of mental development index between children with no or grade 1 peri-intraventricular haemorrhage (PIVH) and children with higher degrees of PIVH.

**Fig 3:**
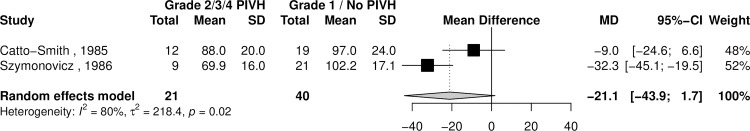
Absolute mean difference of psychomotor development index between children with no or grade 1 peri-intraventricular haemorrhage (PIVH) and children with higher degrees of PIVH.

Our meta-analysis showed no significant difference between the mean Mental Development Index (MDI) when comparing children with no PIVH or Grade 1 PIVH to others with grade 2, 3 or 4 PIVH (Fig 2). Regarding the Psychomotor Development Index (PDI), there was a trend towards lower means in the group with higher degrees of PIVH (2, 3 or 4), but it was not significant (Fig 3).

### Cerebral palsy

**Fig 4:**
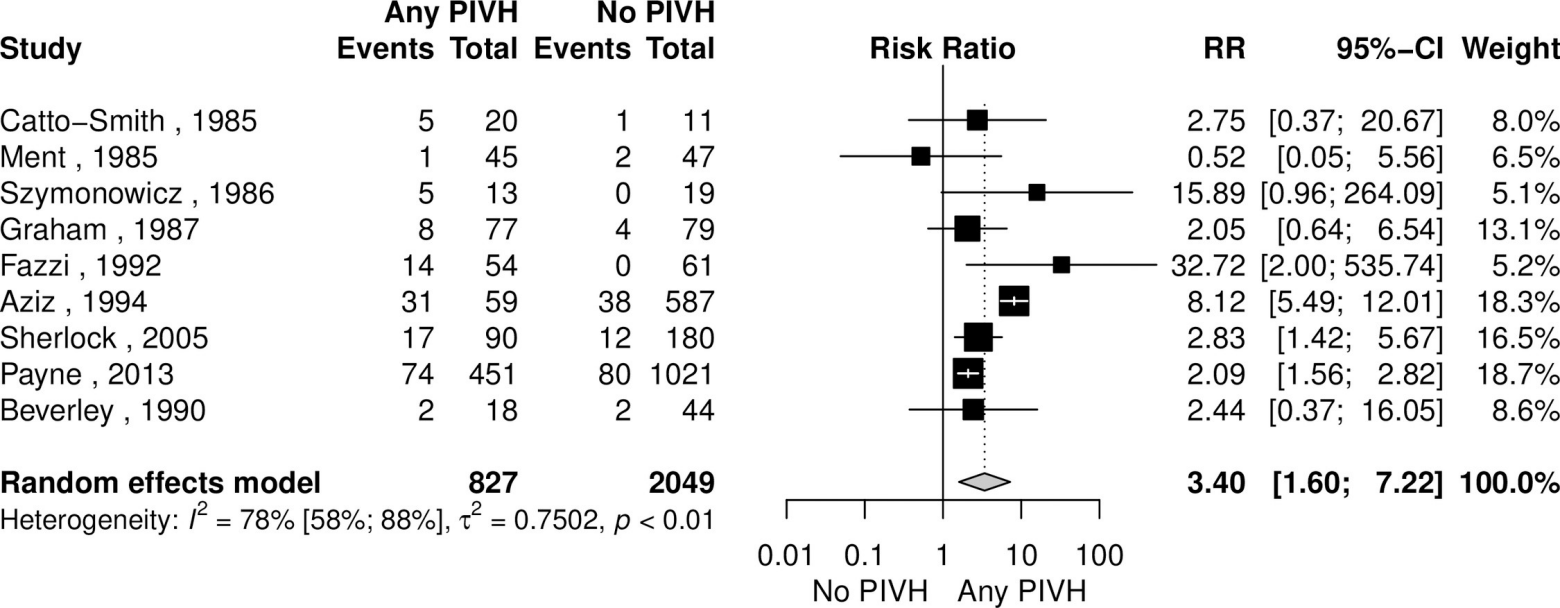
Risk ratio of cerebral palsy comparing children with any peri-intraventricular haemorrhage (PIVH) and those without PIVH.

**Fig 5:**
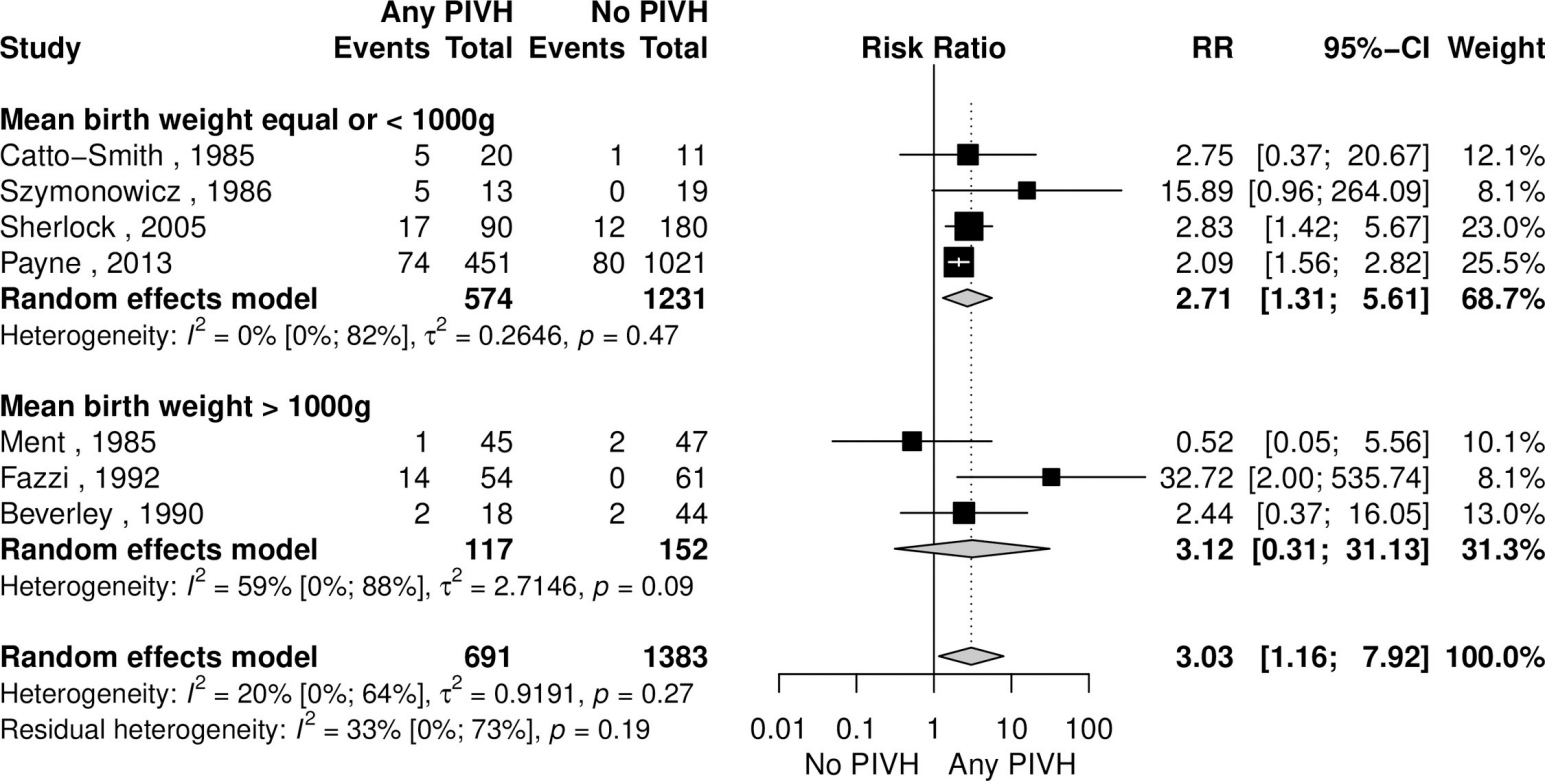
Subgroup analysis of cerebral palsy comparing children with any Peri-Intraventricular Haemorrhage (PIVH) and those without PIVH, by birth weight (BW).

Cerebral palsy was the most commonly reported outcome in studies included in our systematic review. When comparing the group without PIVH and the group with any degree of PIVH, the analysis showed a higher risk of cerebral palsy in neonates with PIVH (Fig 4). For this correlation, a sensitivity analysis was performed excluding articles with zero events, and there was no significant change to the relative risk (RR 2.9 [95% CI 1.5–5.7], I^2^ = 82% [95% CI 63–91%], p < 0.01). An exploratory subgroup analysis was performed dividing studies by the mean cohort birth weight (BW) of included subjects and demonstrated increased risk in the studies with mean BW ≤ 1000 g, a finding which not reproduced with mean cohort BW > 1000g (Fig 5).

For the studies in which it was possible, analysis of mild PIVH (Papile 1 and 2 or equivalent) and severe PIVH (Papile 3 and 4 or equivalent) were tested separately against a group of children with no PIVH. Mild PIVH did not increase the risk of cerebral palsy (4 studies, 2015 subjects, RR 1.99 [95% CI 0.93–4.25], I^2^ = 81% [95% CI 49–93%], p < 0.01), while severe PIVH was associated with a higher risk for this disease (4 studies, 1450 subjects, RR 4.2 [95% CI 1.8–9.9], I^2^ = 15% [0–91%, p < 0.001]. As described in Table 3, our analysis showed increased risk for cerebral palsy in patients presenting any type of PVL compared to those without such abnormalities. Analyzing cystic and non-cystic lesions separately, both conferred increased risk of CP.

**Table 3:** Results for meta-analysis of periventricular leukomalacia for the outcome of cerebral palsy.

Results of meta-analysis of cerebral palsy and periventricular leukomalacia						
Group 1	Group 2	No. Of studies	No of subjects	Pooled RR [95% CI]	p-value	I^2^ (%)
Any PVL	No PVL	2 [4,14]	802	10.63 [1.9–59.5]	<0.001	0
Cystic PVL	No PVL	2 [4,14]	749	19.35 [6.5–46.5]	<0.001	72
Non-cystic PVL	No PVL	2 [4,14]	782	9.08 [5.1–16.1]	<0.001	0

CI, confidence interval; PVL, periventricular leukomalacia; Ref, reference number; RR, Risk Ratio.

### Hearing impairment

Regarding hearing impairment, we found no evidence of increased risk when comparing patients with any degree of PIVH and those without PIVH (5 studies, 2801 subjects, RR 1.20 [0.53–2.69], I^2^ = 0 [95% CI 0–69%], p = 0.61). The results did not change significantly after the sensitivity analysis. The separate analysis between different degrees of PIVH was not possible due to lack of data in the original studies.

When comparing children with cystic PVL and children with no PVL, we found an increased risk of hearing impairment (2 studies, 802 subjects, RR 8.15 [1.45–43.82], I^2^ = 0, p <0.001). There was not enough data to calculate the impact of non-cystic PVL in hearing impairment among preterm infants and sensitivity analysis was not feasible due to the small number of studies.

### Visual impairment

We found no evidence of increased risk of visual impairment in patients presenting any degree of PIVH when compared with those with no PIVH (8 studies, 3057 subjects, RR 2.15 [0.75–6.11], I^2^ = 69% [95% CI 35–85%], p = 0.12). The results did not change significantly after sensitivity analysis. In a similar manner to the analysis of hearing impairment, the analysis between different degrees of PIVH was not possible due to lack of data.

When comparing children with cystic PVL and children with no PVL, we found a significantly increased risk of visual impairment (3 studies, 835 subjects, RR 19.13 [5.23–69.98], I^2^ = 0 [95% CI 0–84%], p < 0.001).

## Discussion

For the outcome of neurodevelopment, our meta-analysis included only the studies which performed the Bayley I test, since there were no sufficient articles featuring Bayley tests II and III. While there was a slight trend towards lower MDI and PDI in children with grades 2/3/4 Papile PIVH, this difference was not statistically significant. However, there are several limitations to this analysis: many articles were lost due to the high heterogeneity of the methods used to assess neurodevelopment and many authors did not present continuous data with its standard deviation. Also, the quality of evidence is weak since data is derived from only three older studies. The article by Hintz [35], a recent study which followed 386 children until 6 to 7 years of age, found a significant risk for cognitive impairment (measured by Full Scale IQ tool) and school-age disability for children with severe PIVH, cystic PVL and cerebellar lesions.

Our findings are comparable with those of a previous meta-analysis [7] which found an increased risk of neurodevelopmental impairment (NDI) in children with PIVH. That study, however, treated neurodevelopmental impairment as a combined outcome of one or more of the following: CP, low mental developmental index, cognitive impairment, visual impairment or hearing impairment. Our analysis further explored this association by separating the combined outcome and our conclusion is that the effect of PIVH in NDI in this previous study may be partially explained by the increased risk of CP in the group with mild or severe PIVH.

The outcome of cerebral palsy was assessed in most articles included in the systematic review and the methods used for its evaluation are usually clinical examination (for 14 studies), Levine classification (for one study) and GMFCS (3 studies). For this outcome, our study corroborated the findings of previous individual studies [28,34,36] which showed an increased risk for CP when the child presented higher degrees of PIVH, nominally Papile grades 3 and 4. However, the association between any degree of PIVH and CP is not seen in studies with a mean BW above 1000 g when subgroup analysis was performed, a finding that may suggest more premature newborns may be more susceptible to the effects of haemorrhage. An obvious bias is that more premature children may have a higher proportion of severe haemorrhage. Our study found no significant increase in the risk of CP in children with mild PIVH (grades 1 and 2). When the assessed exposure was PVL, we found an increased risk of CP in both types of white matter abnormality, cystic and non-cystic, with more evidence for cystic PVL. This finding is important because in some older studies [37–39] only cystic PVL was believed to have significant impact of the brain function. Our study, however, showed that non-cystic PVL has probably almost as much repercussion in the central nervous system as the cystic form. The findings above are extremely relevant for physicians, since the probability of CP and the associated physical limitations are one of the main concerns of the parents when questioning about the preterm child prognosis.

The research group decided to exclude retrospective cohort studies since they may not have standardized both the evaluation of the exposure (imaging studies) and the outcome assessment. Many of those papers are, in fact, a reporting of the experience of a clinical center in a limited amount of time and not studies planned in advance with the methodological rigor expected from a cohort study.

To our knowledge, this is the first meta-analysis which studied the long-term impact of both PVL and PIVH in neurodevelopment of preterm infants separately from other outcomes such as CP, death and disability. One of the main strengths of our study is the separate analysis of all the components usually grouped under the term neurodevelopment, whether motor, cognitive or sensorineural. Also, the separation of patient groups in mild and severe PIVH and cystic and non-cystic PVL, although not possible in all analyses, may have further clarified data of previous individual studies.

The main limitation of this review is the lack of data for PVL in general, especially for the outcome of neurodevelopment. In retrospect, our group believe that this may have been due to the selection criteria which included only studies which performed the initial exam in the first 28 days of life. Many of the studies [40–44] which followed children with PVL performed the imaging studies, usually MRI, at near-term age and were not included in this review. For future research, we believe that another systematic review planned to include more studies of PVL may add to the knowledge in this particular set of patients. However, it is important to state that evidence suggests near-term MRI does not enhance substantively the prediction of late outcomes.

Another major limitation is the small number of studies included in this meta-analysis, which limits the evaluation of publication bias through traditional methods. Since most articles analyzed were published before the year 2000, publication bias should be considered for most of these papers, since older studies are more susceptible to this particular bias [45]. The small number of articles included in the meta-analysis also limits the estimation of heterogeneity across studies, and although statistical methods were used in order to provide a more robust estimation of variance, this remains an important limitation of our study [46]. The search for sources of heterogeneity by subgroup and sensitivity analysis was also limited due to the small percentage of articles which fulfilled inclusion criteria. The results for all meta-analysis are available in S4 File, as well as the complete PRISMA checklist (S5 File). A file containing all complete sheets of included and excluded studies can be found in the supplemental material (S6 File).

## Conclusions

There is evidence to support the hypothesis that children with PIVH and PVL are at increased risk of developing CP, especially when considering children born below 1000 g with PIVH, children with severe PIVH and any type of PVL. Also, children with cystic PVL are probably at increased risk for visual impairment, but evidence is weak. We found no significant difference between means of cognitive and psychomotor scales for children with and without PIVH, but evidence is poor and additional studies may further explore this outcome.

## Supporting information

S1 File Complete search strategy. (PDF)

S2 File Modified Newcastle-Ottawa quality assessment scale cohort studies. (PDF)

S3 File Results of the modified Newcastle-Ottawa scale. (PDF)

S4 File Results of all meta-analysis performed. (PDF)

S5 File PRISMA statement checklist. (PDF)

S6 File Exclusion tables for study reproduction. (RAR)
