# Supplementary material for: Impact of peri-intraventricular haemorrhage and periventricular leukomalacia in the neurodevelopment of preterms: A systematic review and meta-analysis
Source: PLoS One. 2019 Oct 10;14(10):e0223427. doi: 10.1371/journal.pone.0223427 (PMC6786801; doi:10.1371/journal.pone.0223427)
Supplement: S1 File — (PDF) [file pone.0223427.s001.pdf]

SEARCH STRATEGY

PubMed:

(premature OR prematurity OR preterm) AND ((“periventricular leukomalacia” OR “white matter lesion”) OR (“brain hemorrhage” OR “brain haemorrhage”)) AND ((“cerebral palsy” OR neurodevelopment OR “motor assessment” OR “cognitive development” OR "developmental disability" OR “neurodevelopmental outcome”) OR (epilepsy OR seizures) OR blindness OR deafness)

= 574 references

Embase:

('premature'/exp OR 'prematurity'/exp OR preterm) AND ('periventricular leukomalacia'/exp OR 'white matter lesion'/exp OR 'intraventricular hemorrhage' OR 'intraventricular haemorrhage') AND ('cerebral palsy'/exp OR 'neurodevelopment'/exp OR 'motor assessment' OR 'cognitive development'/exp OR 'developmental disability' OR 'neurodevelopmental outcome'/exp OR 'epilepsy'/exp OR 'seizures'/exp OR 'blindness'/exp OR 'deafness'/exp) AND 'human'/exp

= 521 references

SciELO:

(prematuro) OR (prematuridade) OR (pretermo) AND (leucomalácia periventricular) OR (lesão de substância branca) OR (hemorragia cerebral) AND (paralisia cerebral) OR (neurodesenvolvimento) OR (avaliação motora) OR (desenvolvimento cognitivo) OR (anormalidade do desenvolvimento) OR (desfechos de desenvolvimento) OR (epilepsia) OR (convulsões) OR (cegueira) OR (surdez)

= 6 references

LILACS:

(tw:(premature)) OR (tw:(prematurity)) OR (tw:(preterm)) AND (tw:(periventricular leukomalacia)) OR (tw:(white matter lesion)) OR (tw:(brain hemorrhage)) OR (tw:(brain haemorrhage)) AND (tw:(cerebral palsy)) OR (tw:(neurodevelopment)) OR (tw:(motor assessment)) OR (tw:(cognitive development)) OR (tw:(developmental disability)) OR (tw:(neurodevelopmental outcome)) OR (tw:(epilepsy)) OR (tw:(seizures)) OR (tw:(blindness)) OR (tw:(deafness))

= 483 references

Cochrane databases:

(premature or prematurity or preterm) and (("periventricular leukomalacia" or "white matter lesion") or ("brain hemorrhage" or "brain haemorrhage")) and (("cerebral palsy" or neurodevelopment or "motor assessment" or "cognitive development" or "developmental disability" or "neurodevelopmental outcome") or (epilepsy or seizures) or blindness or deafness)

= 235 references
