# Supplementary material for: Impact of peri-intraventricular haemorrhage and periventricular leukomalacia in the neurodevelopment of preterms: A systematic review and meta-analysis
Source: PLoS One. 2019 Oct 10;14(10):e0223427. doi: 10.1371/journal.pone.0223427 (PMC6786801; doi:10.1371/journal.pone.0223427)
Supplement: S2 File — (PDF) [file pone.0223427.s002.pdf]

Modified Newcastle-Ottawa quality assessment scale
cohort studies

No.	Criterion	Decision rule	Score (*=1, no*=0)
SELECTION			
1	Representativeness of the exposed cohort	a) Consecutive eligible participants were selected, participants were randomly selected, or all participants were invited to participate from the source population*<br>b) Not satisfying requirements in part (a), or not stated.	
2	Selection of the non-exposed cohort	a) Selected from the same source population*<br>b) Selected from a different source population<br>c) No description	
3	Ascertainment of exposure	a) Structured injury data (e.g. record completed by medical staff)*<br>b) Structured interview*<br>c) Written self-report<br>d) No description	
4	Demonstration that outcome of interest was not present at the start of the study	a) Yes*<br>b) No or not explicitly stated	
COMPARABILITY			
1	Comparability of cohorts on the basis of the design or analysis	a) Study controls for age*<br>b) Study controls for birth weight*<br>*Note*: Exposed and non-exposed individuals must be matched in the design and/or confounders must be adjusted for in the analysis. Alone statements of no differences between groups or that differences were not statistically significant are not sufficient.	
OUTCOME			
1	Assessment of outcome	a) Independent or blind assessment stated, or confirmation of the outcome by reference to secure records (e.g. imaging, structured injury data, etc.)*<br>b) record linkage (e.g. identified through ICD codes on database records)*<br>c) Self-report with no reference to original structured injury data or imaging<br>d) No description	
2	Was follow-up long enough for outcomes to occur?	a) Yes (≥12 months)*<br>b) No (<3 months)	
3	Adequacy of follow up of cohorts	a) Complete follow up – all participants accounted for*<br>b) Subjects lost to follow up unlikely to introduce bias (<20% lost to follow up, or description provided of those lost*)<br>c) Follow up rate <85% and no description of those lost provided<br>d) No statement	
SCORE			
