# Supplementary material for: Impact of peri-intraventricular haemorrhage and periventricular leukomalacia in the neurodevelopment of preterms: A systematic review and meta-analysis
Source: PLoS One. 2019 Oct 10;14(10):e0223427. doi: 10.1371/journal.pone.0223427 (PMC6786801; doi:10.1371/journal.pone.0223427)
Supplement: S3 File — (PDF) [file pone.0223427.s003.pdf]

Results of the modified Newcastle-Ottawa scale

Nº	Author	Selection	Comparability	Outcome	Total	Risk of Bias
1	Catto-Smith	4	0	2	6	Moderate
2	Ment	4	0	3	7	Moderate
3	Szymonowicz	4	0	2	6	Moderate
4	Graham	4	0	3	7	Moderate
5	Salomon	4	0	2	6	Moderate
6	Nwaesei	4	0	2	6	Moderate
7	Bennett	3	0	3	6	Moderate
8	Beverley	3	0	2	5	High
9	van de Bor	2	0	3	5	High
10	Fazzi	4	0	3	7	Moderate
11	Ikonen	4	0	2	6	Moderate
12	van de Bor	4	2	2	8	Low
13	Roth	4	0	3	7	Moderate
14	Aziz	4	0	2	6	Moderate
15	Fawer	3	0	3	6	Moderate
16	Vohr	4	0	1	5	High
17	Sherlock	4	0	3	7	Moderate
18	Dyet	4	1	2	7	Moderate
19	Vollmer	4	0	3	7	Moderate
20	Locatelli	4	0	2	6	Moderate
21	van Wezel-Meijler	3	0	1	4	High
22	Klebermass-Schrehof	3	0	2	5	High
23	Payne	4	1	3	8	Low
24	Hintz	4	1	3	8	Low
