# Supplementary material for: Impact of peri-intraventricular haemorrhage and periventricular leukomalacia in the neurodevelopment of preterms: A systematic review and meta-analysis
Source: PLoS One. 2019 Oct 10;14(10):e0223427. doi: 10.1371/journal.pone.0223427 (PMC6786801; doi:10.1371/journal.pone.0223427)

RESULTS OF ALL META-ANALYSIS PERFORMED

1) Exposure: Peri-intraventricular haemorrhage / Outcome: Cerebral palsy.

a) Relative risk of cerebral palsy in children with PIVH Grades 3 and 4 compared to children with no PIVH.

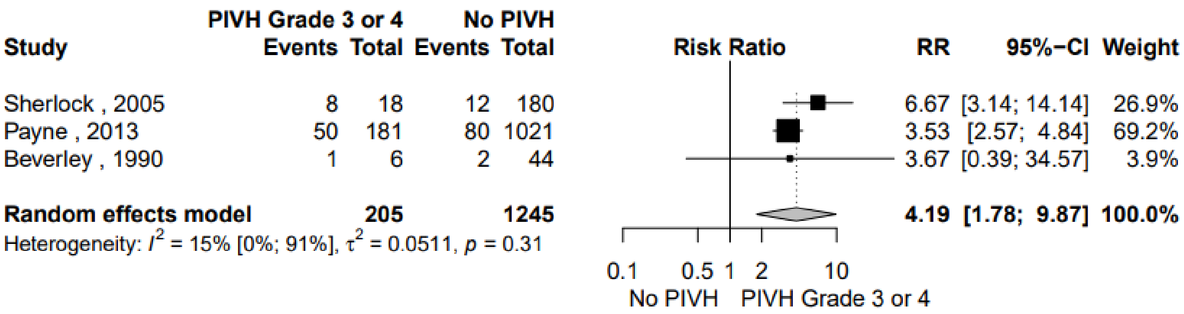

b) Relative risk of cerebral palsy in children with PIVH Grades 1 and 2 compared to children with no PIVH.

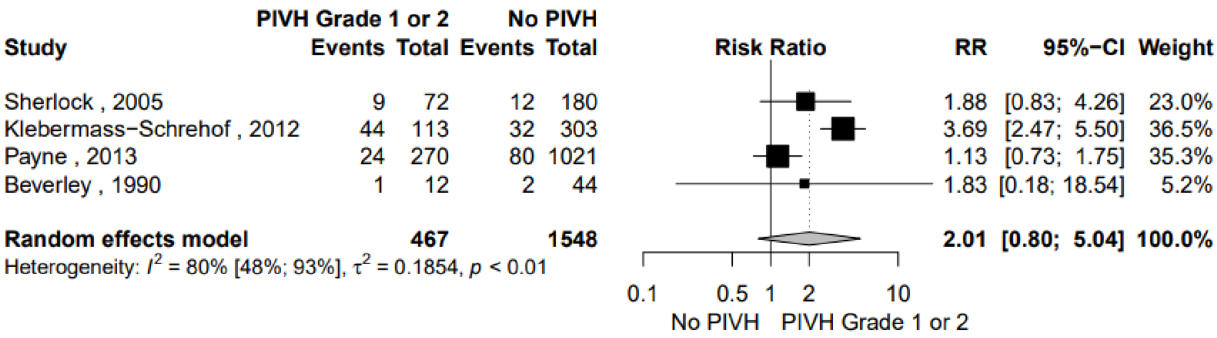

c) Relative risk of cerebral palsy in children with any degree of PIVH compared to children with no PIVH, followed by funnel plot analysis. Followed by sensitivity analysis.

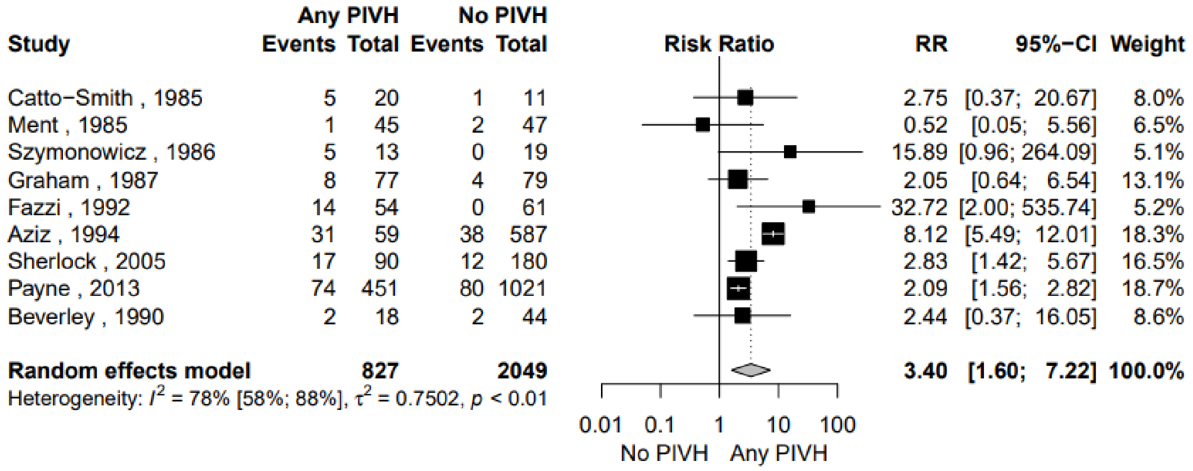

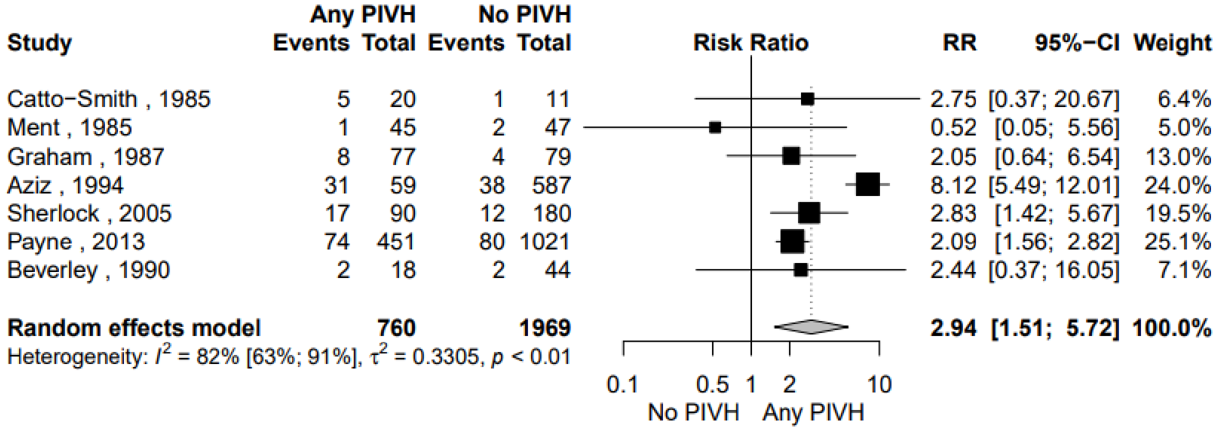

d) Relative risk of cerebral palsy in children with any degree of PIVH compared to children with no PIVH separated in subgroups by birth weight. Followed by sensitivity analysis.

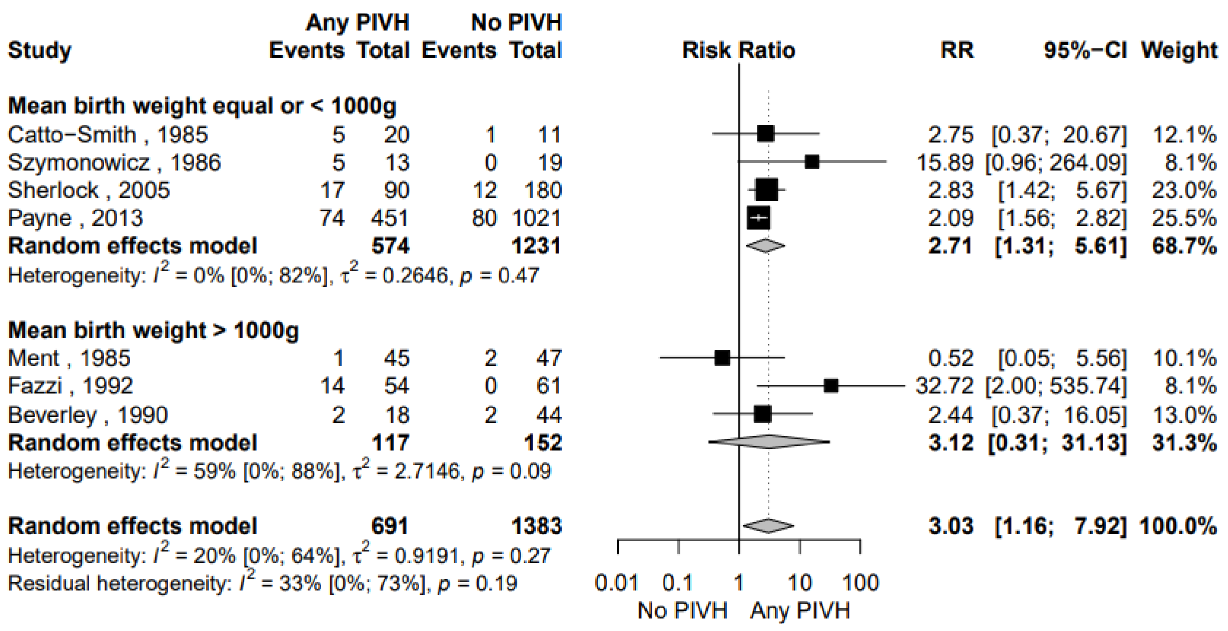

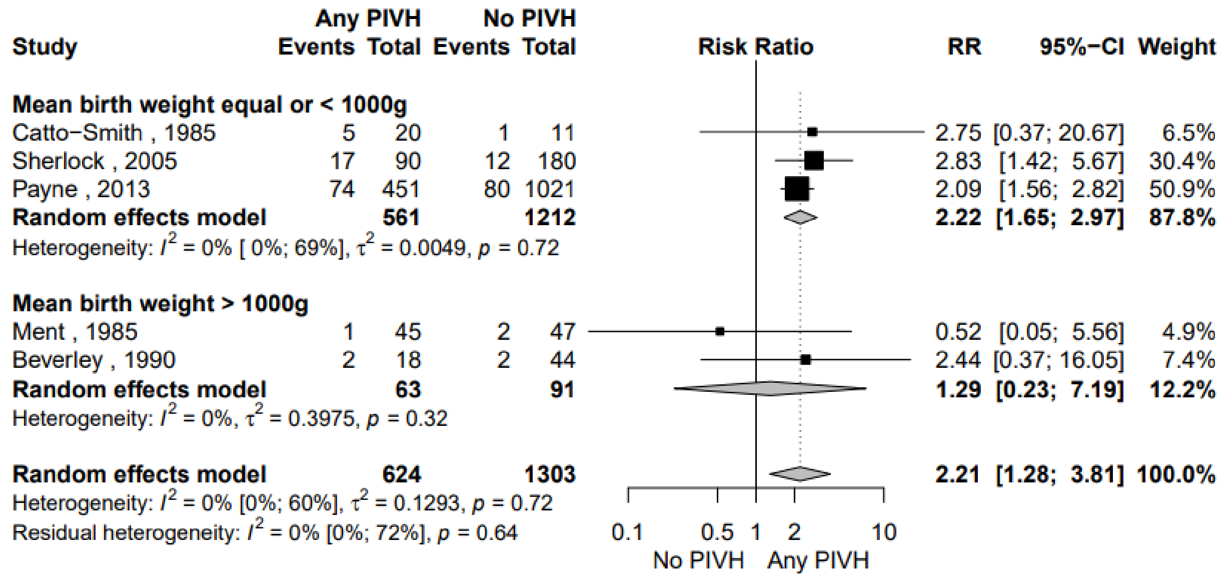

2) **Exposure: Peri-intraventricular haemorrhage / Outcome: Visual impairment.**

a) Relative risk of visual impairment in children with any degree of PIVH compared to children with no PIVH. Followed by sensitivity analysis.

Study	Any PIVH Events	Any PIVH Total	No PIVH Events	No PIVH Total	RR	95%−CI	Weight
Catto−Smith , 1985	1	20	1	11	0.55	[0.04; 7.96]	8.0%
Szymonowicz , 1986	1	13	0	19	4.33	[0.19; 98.57]	6.4%
Graham , 1987	2	77	1	79	2.05	[0.19; 22.17]	9.4%
van de Bor , 1993	1	67	4	234	0.87	[0.10; 7.68]	10.5%
Roth , 1993	1	81	1	112	1.38	[0.09; 21.78]	7.7%
Aziz , 1994	11	59	6	587	18.24	[7.00; 47.54]	20.4%
Fawer , 1995	3	32	16	194	1.14	[0.35; 3.68]	18.3%
Payne , 2013	5	451	10	1021	1.13	[0.39; 3.29]	19.4%
**Random effects model**		**800**		**2257**	**2.15**	**[0.75; 6.11]**	**100.0%**

Heterogeneity: $I^2 = 69\%$ [35%; 85%], $\tau^2 = 0.8048$, $p < 0.01$

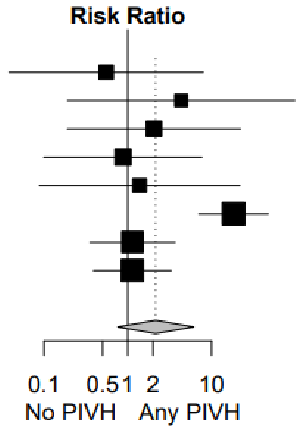

Study	Any PIVH Events	Any PIVH Total	No PIVH Events	No PIVH Total	RR	95%−CI	Weight
Catto−Smith , 1985	1	20	1	11	0.55	[0.04; 7.96]	8.9%
Graham , 1987	2	77	1	79	2.05	[0.19; 22.17]	10.3%
van de Bor , 1993	1	67	4	234	0.87	[0.10; 7.68]	11.5%
Roth , 1993	1	81	1	112	1.38	[0.09; 21.78]	8.5%
Aziz , 1994	11	59	6	587	18.24	[7.00; 47.54]	21.3%
Fawer , 1995	3	32	16	194	1.14	[0.35; 3.68]	19.3%
Payne , 2013	5	451	10	1021	1.13	[0.39; 3.29]	20.3%
**Random effects model**		**787**		**2238**	**2.02**	**[0.62; 6.59]**	**100.0%**

Heterogeneity: $I^2 = 73\%$ [43%; 88%], $\tau^2 = 0.9184$, $p < 0.01$

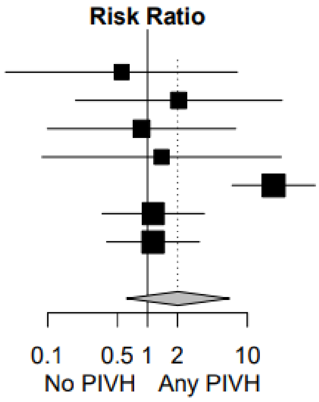

3) Exposure: Peri-intraventricular haemorrhage / Outcome: Hearing impairment.

a) Relative risk of hearing impairment in children with any degree of PIVH compared to children with no PIVH. Followed by sensitivity analysis.

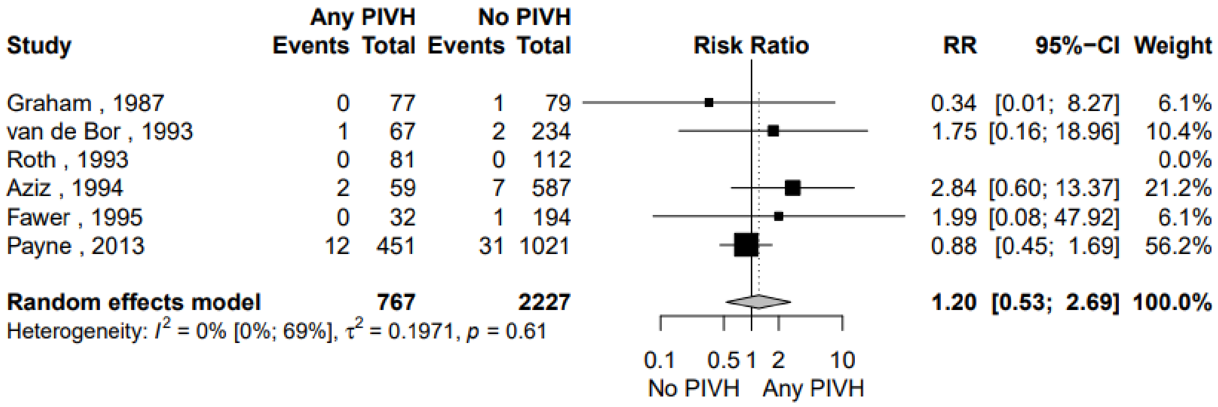

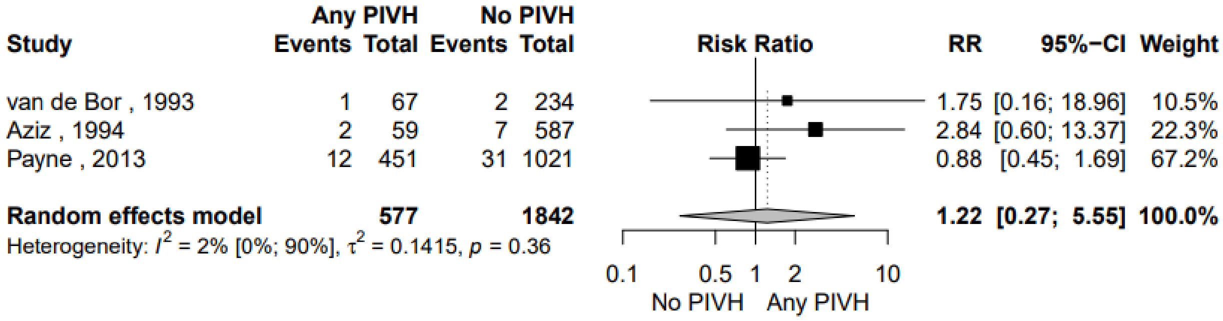

4) Exposure: Peri-intraventricular haemorrhage / Outcome: Bayley I Scale

a) Mean difference in Mental Development Index (Bayley I Scale) between children with PIVH grades 2, 3 and 4 compared to children with grade 1 and no PIVH.

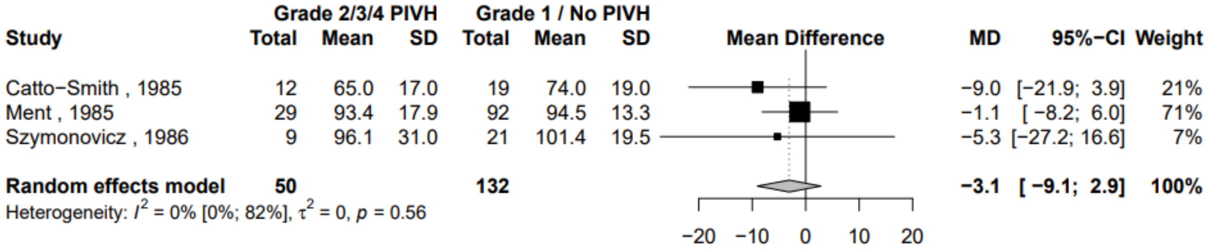

b) Mean difference in Psychomotor Development Index (Bayley I Scale) between children with PIVH grades 2, 3 and 4 compared to children with grade 1 and no PIVH.

Study	Grade 2/3/4 PIVH Total	Mean	SD	Grade 1 / No PIVH Total	Mean	SD	MD	95%−CI	Weight
Catto−Smith , 1985	12	88.0	20.0	19	97.0	24.0	−9.0	[−24.6; 6.6]	48%
Szymonovicz , 1986	9	69.9	16.0	21	102.2	17.1	−32.3	[−45.1; −19.5]	52%
**Random effects model**	**21**			**40**			**−21.1**	**[−43.9; 1.7]**	**100%**

Heterogeneity: $I^2 = 80\%$, $\tau^2 = 218.4$, $p = 0.02$

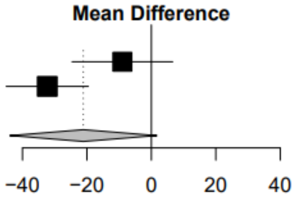

5) Exposure: Periventricular leukomalacia / Outcome: Cerebral palsy

a) Relative risk of cerebral palsy in children with any form of PVL compared to children with no PVL.

Study	Any PVL Events	Total	No PVL Events	Total	RR	95%−CI	Weight
Graham , 1987	10	37	2	119	16.08	[3.69; 70.13]	9.7%
Aziz , 1994	21	36	35	610	10.17	[6.65; 15.53]	90.3%
**Random effects model**		**73**		**729**	**10.63**	**[1.90; 59.54]**	**100.0%**

Heterogeneity: $I^2 = 0\%$, $\tau^2 = 0.0154$, $p = 0.56$

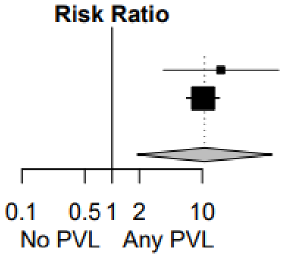

b) Relative risk of cerebral palsy in children with cystic PVL compared to children with no PVL.

Study	Cystic PVL Events	Total	No PVL Events	Total	RR	95%−CI	Weight
Graham , 1987	8	13	2	119	36.62	[8.68; 154.52]	30.8%
Aziz , 1994	5	7	35	610	12.45	[7.05; 21.98]	69.2%
**Random effects model**		**20**		**729**	**17.35**	**[6.48; 46.47]**	**100.0%**

Heterogeneity: $I^2 = 46\%$, $\tau^2 = 0.2809$, $p = 0.17$

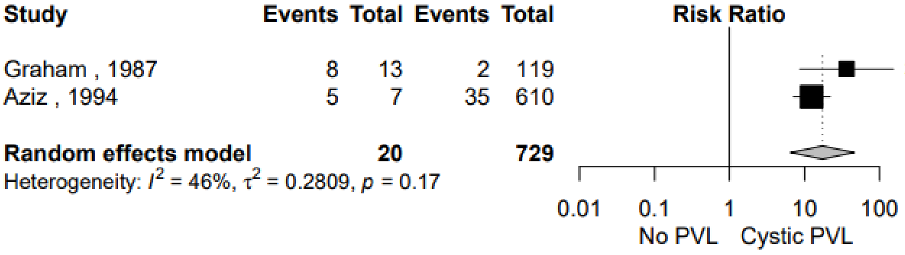

c) Relative risk of cerebral palsy in children with non-cystic PVL compared to children with no PVL.

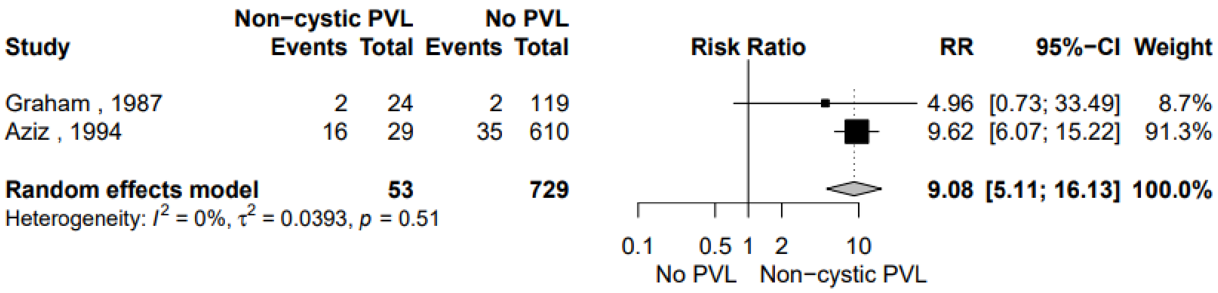

6) Exposure: Periventricular leukomalacia / Outcome: Visual impairment

a) Relative risk of visual impairment in children with cystic PVL compared to children with no PVL. Sensitivity analysis was not possible.

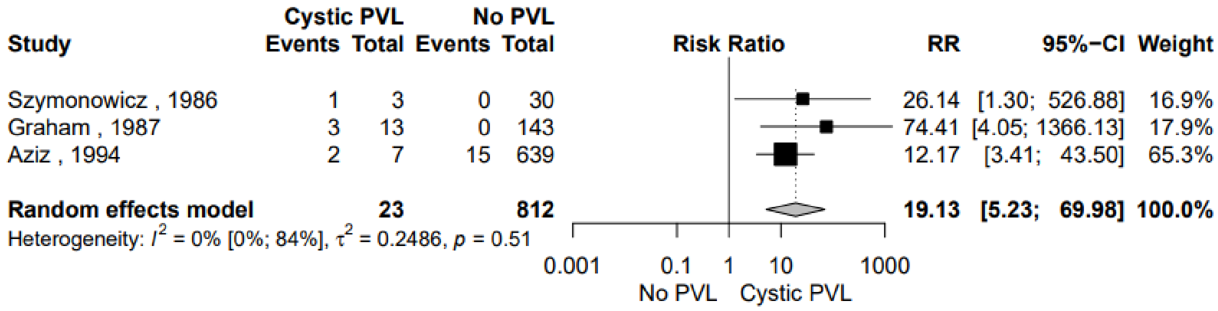

7) Exposure: Periventricular leukomalacia / Outcome: Hearing impairment

a) Relative risk of hearing impairment in children with cystic PVL compared to children with no PVL. Sensitivity analysis was not possible.

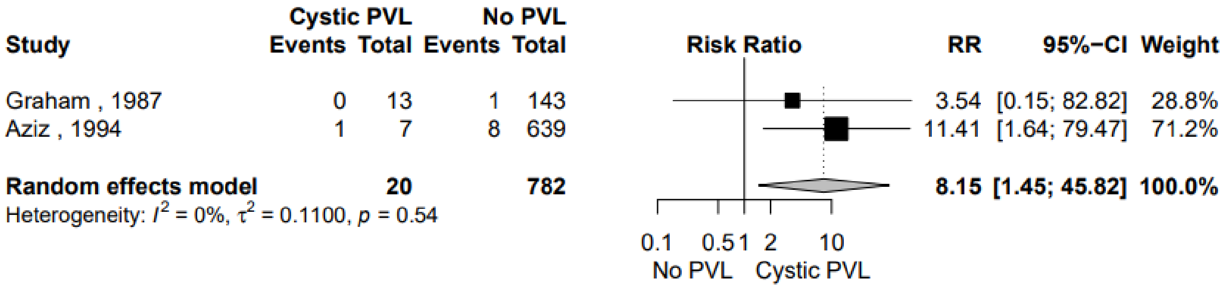

Supplement: S4 File — (PDF) [file pone.0223427.s004.pdf]
